# Psychometric properties of the Arabic version of the 10-item State Shame and Guilt Scale

**DOI:** 10.3389/fpsyg.2026.1807388

**Published:** 2026-05-13

**Authors:** Alain Chbeir, Nelly Kheir, Souheil Hallit, Roudina Waleed, Rayan Ali, Shahd Jalal, Ayah Al-Zubi, Nouran Omar El Said, Nada Kassem, Abir Sarray El Dine, Muna Barakat, Bassam Abdul Rasool Hassan, Feten Fekih-Romdhane, Sahar Obeid, Diana Malaeb

**Affiliations:** 1 School of Medicine and Medical Sciences, Holy Spirit University of Kaslik, Jounieh, Lebanon; 2 Faculty of Medicine, Paris-Saclay University, Le Kremlin-Bicêtre, France; 3 College of Pharmacy, Gulf Medical University, Ajman, United Arab Emirates; 4 Faculty of Pharmacy, Future University in Egypt, Cairo, Egypt; 5 School of Pharmacy, Lebanese International University, Beirut, Lebanon; 6 Department of Biomedical Sciences, School of Arts and Sciences, Lebanese International University, Beirut, Lebanon; 7 Department of Clinical Pharmacy and Therapeutics, School of Pharmacy, Applied Science Private University, Amman, Jordan; 8 School of Pharmacy, Al Rafidain University College, Baghdad, Iraq; 9 The Tunisian Center of Early Intervention in Psychosis, Department of Psychiatry “Ibn Omrane”, Razi Hospital, Manouba, Tunisia; 10 Faculty of Medicine of Tunis, Tunis El Manar University, Tunis, Tunisia; 11 Department of Psychology and Education, School of Arts and Sciences, Lebanese American University, Jbeil, Lebanon

**Keywords:** Arabic version, concurrent validity, measurement invariance, State Shame and Guilt Scale, validation

## Abstract

**Background:**

Guilt and shame are self-conscious emotions that affect behavior and mental health, with meanings that vary across cultures. In Arab society, shame is more related to social and moral values, while guilt is related to relational obligations. To date, no validated tool exists to assess both emotions in Arab populations, leading professionals to misrepresent how Arabs perceive guilt and shame. This limits clinical and research accuracy in understanding their relationship with psychological distress and other factors. Therefore, the present study aims to translate and validate the 10-Item State Shame and Guilt Scale into an Arabic version to provide an accurate tool for assessing guilt and shame among Arabs.

**Methods:**

611 adults from Lebanon, Jordan, Egypt, and Iraq were recruited via an anonymous self-administered Google Forms survey. All questions were mandatory, resulting in no missing data. Confirmatory Factor Analysis (CFA) was conducted using RStudio (R version 4.5.2), with the “lavaan” and “SemTools” packages, applying the Weighted Least Squares Mean and Variance Adjusted (WLSMV) estimator appropriate for ordinal data to perform on the full sample.

**Result:**

The CFA showed that the two-factor model of the shame and guilt scale demonstrated acceptable to good fit indices (X^2^ (34) = 175.62, *p* < 0.001; RMSEA = 0.082, SRMR = 0.059; CFI = 0.993, TLI = 0.991). Internal consistency was excellent for both shame (McDonald’s *ω* = 0.86; Cronbach’s *α* = 0.87) and guilt (ω = 0.87; α = 0.87), with satisfactory convergent validity (AVE = 0.75). Configural, metric, and scalar invariance were supported with no significant differences observed between males and females or across countries in shame or guilt scores. Our results also show that both shame and guilt scores were significantly and moderately associated with external and internal shame, psychological distress, and perfectionism, supporting the scale’s validity in capturing self-conscious emotional processes related to mental health outcomes.

**Conclusion:**

The Arabic version of the 10-item State Shame and Guilt Scale turned out to be a reliable, valid, and cost-effective psychometric scale to assess guilt and shame in the Arab culture. It shows meaningful associations with mental health outcomes, giving a valuable tool for researchers and clinicians to study self-conscious emotions and their impact on Arabic-speaking populations.

## Introduction

Guilt and shame are self-conscious emotions, unlike basic emotions such as fear, anger, or sadness. These emotions are not defined by triggering events; rather, they depend on advanced cognitive processes, including self-awareness and evaluation of one’s own actions (Miceli and Castelfranchi, 2018; Oh and Cho, 2023). Individuals rely on these emotions to form a sense of self, judge their moral standards, and regulate their behavior in social situations (Oh and Cho, 2023). Studies have shown that guilt and shame do not function in the same way. They produce different behavioral responses and have a long-term impact on mental health (Fromson, 2006; Miceli and Castelfranchi, 2018). Tangney and Dearing have demonstrated that guilt and shame stem from the same negative situation. Thus, the critical distinction between the two emotions does not lie in the external circumstances themselves, but in the individual interpretation of the event (Tangney and Dearing, 2003).

Shame is the emotion experienced by the self as it becomes the subject of observation and judgment (Tangney et al., 1996). It is defined by a negative evaluation, as it targets the whole identity and not a particular act (Miceli and Castelfranchi, 2018; Tangney et al., 1996). A person experiencing shame feels “not good enough”, “defective”, “insufficient”, or simply says “I am bad”. Shame may be experienced in two forms: internally, through intense self-reproach, or externally, through the belief that others are criticizing them (Parker and Thomas, 2009; Sheehy et al., 2019). This emotion can cause significant distress and is related to a spectrum of psychological problems, such as depression, anxiety, eating disorders, and more (Tangney, 2001). People may often use maladaptive strategies to cope with shame, including overthinking their mistakes, pulling away from others, becoming overly sensitive to criticism, avoiding triggering situations, or hiding what they see as flaws (Cheung et al., 2004; Gilbert et al., 1994).

Shame is conceptualized not only as a stable, fixed personality trait but also as a dynamic emotional state that depends on context and can be induced in controlled settings. For example, an experimental study showed that the Autobiographical Emotional Memory Task (AEMT) was found to induce an acute increase in shame (Fink-Lamotte et al., 2023). This supports that shame can be provoked in experimental settings by asking individuals to recall personally meaningful experiences. Similarly, an experimental study using thought-action fusion (TAF) demonstrates that exposure to morally disturbing intrusive thoughts can significantly increase the level of state shame compared to control conditions (Hansmeier et al., 2023). Therefore, shame is a dynamic emotional state that varies with the situation and its interpretation. Recognizing this distinction in experimental research is important, where sensitive measures are needed to detect short-term emotional changes and to assess how shame varies within individuals and in response to interventions.

Guilt is defined as an emotion that focuses on actions rather than the whole identity (Miceli and Castelfranchi, 2018; Parker and Thomas, 2009). It is felt when individuals judges their conduct as violating their moral principles (Miceli and Castelfranchi, 2018). When feeling guilty, individuals view a specific behavior or decision as wrong (“I did something bad”), which produces psychological discomfort, self-blame, regret, and an ongoing preoccupation with the event (Shi et al., 2021; Tangney, 2001). Rather than driving avoidance, guilt encourages accountability and corrective behavior. It is important to note that guilt is conceptualized and measured differently in the literature, with some approaches emphasizing its adaptive and prosocial functions, while others focus on its maladaptive or clinical aspects. The present study adopts a perspective that emphasizes the adaptive and self-evaluative functions of guilt. This leads people to admit fault, apologize, and seek efforts to repair the harm caused (Bastin et al., 2016; Miceli and Castelfranchi, 2018). Therefore, guilt affects moral evaluation and does not disrupt one’s identity. Hence, despite the distressing nature of this emotion, guilt tends to result in more adaptive and functional responses than shame, and it carries lower risks of chronic psychological disorders (Cheung et al., 2004; Tangney et al., 2007).

Given the central role of guilt and shame in regulating the moral emotions and in psychopathology, many studies have established an association between these emotions and mental health conditions (Gilbert et al., 1994; Parker and Thomas, 2009; Tangney et al., 1996). Research on 1,266 teachers has shown an association with burnout (Figueiredo-Ferraz et al., 2021), and another study found that guilt and anxiety are strongly interrelated (Miceli and Castelfranchi, 2018; Westerink, 2021). Cross-sectional studies have also reported that exposure to trauma is strongly connected with elevated levels of these emotions, which are highly prevalent in those individuals (Miller et al., 2013; Murray, 2018). Additionally, there is evidence that uncontrolled or excessive emotions are closely linked to major depressive disorder and to the exacerbation of its symptoms (Alexander et al., 1999; Tangney et al., 1992).

For instance, psychological distress usually includes the emotional suffering, which consists of depression, anxiety, social withdrawal, and impairments in daily functioning. Different meta-analyses have demonstrated that shame and guilt are contributing components to psychological distress. Indeed, shame has a higher correlation with depression than guilt (Kim et al., 2011), as well as with suicidal behavior, non-suicidal self-injury (Sheehy et al., 2019), symptoms of eating disorders (Nechita et al., 2021), and all trauma-related disturbances involving difficulty organizing oneself, avoidance, and hyperarousal (e.g., not being aware of your environment) (Murray, 2018; Oasi et al., 2025; Saraiya and Lopez-Castro, 2016). Guilt, on the other hand, although it promotes behaviors that are reparative toward other people (Miceli and Castelfranchi, 2018), can take the form of maladaptive guilt (Kim et al., 2011). This type of guilt, such as generalized guilt or hyper-responsible guilt, is associated with depressive symptoms (Kim et al., 2011), trauma intrusions and prolonged rumination, which shows that guilt can also contribute to psychological distress (Bravo et al., 2020).

Additionally, perfectionism is a complex set of traits that includes the pursuit of high standards, a focus on mistakes, and on other people’s opinions (Gäde et al., 2017). According to current evidence, people who display maladaptive perfectionist behavior are likely to experience greater shame when they view their failures as a reflection of themselves (Barritt, 2017; Stoeber et al., 2007). Further research has shown that people who have pre-made perfectionistic standards tend to feel guilty more than others after making an error in their behavior, which can become distressing when guilt is combined with perfectionism-related anxiety (Barritt, 2017; Stoeber et al., 2007). Overall, the degree of shame and guilt that an individual experiences as a result of a perceived failure can be significantly affected by the extent to which perfectionism is present in the individual (Stoeber et al., 2007).

Accordingly, assessing guilt and shame as closely related yet distinct entities necessitates the development and validation of reliable measurement tools. Currently, a wide range of self-report instruments has been developed to evaluate these emotions. These tools are classified into three main categories: adjective-based, scenario-based, and statement-based measures. Firstly, the adjective-based measures include the Internalized Shame Scale (ISS) and the Personal Feelings Questionnaire-2 (PFQ-2). These scales assess the general frequency of guilt and shame but have a limitation in addressing the situational and behavioral differences of these emotions (Cook, 2013; Harder et al., 1993; Tzelepi et al., 2022). Secondly, there are the scenario-based measures, notably the Guilt and Shame Proneness Scale (GASP) and the Tests of Self-Conscious Affect (TOSCA). The main objective here is the study of guilt and shame in an imagined situation, to see how people would think, feel, and act. However, the use of these scales is time-consuming and can mix emotional and behavioral responses, making them harder to use in large-scale studies (Alabèrnia-Segura et al., 2022; Tzelepi et al., 2022; Watson et al., 2017). Thirdly, statement-based measures contain the State Shame and Guilt Scale, the Adolescent Shame-Proneness Scale, and the Brief Shame and Guilt Questionnaire for Children, abbreviated by SSGS, ASPS, and BSGQ-C. This category requires participants to indicate their agreement with statements reflecting experiences of guilt and shame, including current or “state” emotions. Even though these tools capture the immediate emotional response, they may be limited by population specificity (like BSGQ-C) or focus on a single emotion (like shame in ASPS) (Cavalera et al., 2017; Novin and Rieffe, 2015; Simonds et al., 2016). Among the most widely used scales to evaluate both shame and guilt is the 10-item Guilt and Shame scale (Marschall et al., 1994). This scale was initially created in English (Marschall et al., 1994), and both subscales have demonstrated good internal consistency, with Cronbach’s *α* coefficients ranging from 0.82 to 0.89. Afterward, this scale has been validated in Greek (Tzelepi et al., 2022), Italian (Cavalera et al., 2017), and Tamil (Lebastchi, 2016), with data showing consistency with the original scale. For example, in the Italian version, the RMSEA of 0.067 was in accordance with the cutoff value of 0.08 (Hu and Bentler, 1999), indicating that invariance was supported at all levels in this translated version. This suggests the translated version has an acceptable fit; if applied to the entire population, it would yield similar results. Thus, the scale measures the same construct similarly across groups, making its units comparable between groups. This was also proved by the SRMR measured in the Italian translation (0.045), also in accordance with the cut-off value (Marsh et al., 2013), supporting the two-factor structure of this scale and further proving that if it is used to measure shame and guilt in different populations, it yields comparable results. As a result, the SSGS has been demonstrated as an effective instrument for measuring transient emotions of guilt and shame in both research and clinical contexts.

A cross-cultural study noted that guilt and shame are strongly shaped by cultural values, social structures, and moral norms (Bedford and Hwang, 2003). This research primarily examined two contrasting cultural environments: the individualistic values of Western society and the relationship-oriented values of Asian cultures (including China and Japan). The primary distinction between the two cultures is that, in Western cultures, guilt relates most to one’s personal responsibility for one’s actions, whereas shame relates more directly to one’s individual identity and reputation (Bedford and Hwang, 2003). In Asia, guilt concerns fulfilling obligations and duties to others, and shame serves to guide individuals toward appropriate actions and to preserve the collective interests of their communities through social harmony. Because of these cultural differences, the instruments designed to measure the feelings of guilt and shame in one cultural setting may not accurately measure them in other cultures (Bedford and Hwang, 2003). Accordingly, in the Arab region, the concepts of guilt and shame may also have distinct characteristics compared to those in Western countries (Fekih-Romdhane et al., 2023a).

In Arab culture, shame is not limited to an emotional experience but also carries strong social and moral meanings. This reflects that shame is the same emotion across cultures, but it may be used and emphasized differently in social settings. In this context, shame occupies a central place in the social life of Arabs and is commonly known by various terms, such as “ḥayāʾ”, “ʿayb,” and “khajal” (Al Jallad, 2010). Those different words refer all to shame, but each has its own nuances, from having a sense of inner moral discipline to fearing exposure in front of others, due to breaking a common social or moral rule. Research done in the area has shown that the idea of shame is rooted in family life, gender expectations, concepts of honor, and that a person can feel shame even when no one is watching (Al Jallad, 2010). Because shame is so closely tied to culture, it is very noticeable in an Arab’s daily life but can also look very different depending on the situation or social context. For guilt, it is usually called “al-dhanb,” and it is understood by recognizing that one’s actions were wrong (El Alaoui et al., 2017). A study examining Saudi women showed that the guilt they experienced is more linked to how their actions affect other people (family, friends, and society) rather than just focusing on themselves (El Alaoui et al., 2017). This reflects the social values of Arab culture, where guilt encourages people to make amends. It not only guides personal morals but also helps maintain relationships and social bonds.

Moreover, a study comparing the United Arab Emirates (UAE) and Ireland found that shame and guilt function differently across these cultures (Grey et al., 2018). The data from this study revealed that in the UAE, women report higher levels of both emotions, and shame is strongly associated with a negative self-evaluation. In Ireland, instead of criticizing themselves, they deal with shame by simply avoiding people and skipping social interactions. For instance, UAE women’s shame is self-focused and evaluative, unlike Irish women’s shame, where it is action-focused and avoidant (Grey et al., 2018). This difference further supports the idea that shame and guilt are not experienced in the same way across all cultures. As such, when using a scale developed for a Western sample and applying it to Arab culture without careful translation and validation, the experience of guilt and shame of the Arab society may be misrepresented. This highlights the necessity of having an Arabic validated scale that accurately captures these feelings. However, the current literature lacks culturally and linguistically appropriate tools to perform this evaluation. This gap can be addressed by a validated Arabic version of the 10-item SSGS, which researchers and clinicians need to access a useful and culturally appropriate measurement tool. Thereby, it will advance research, promote evidence-based clinical practices, and facilitate therapy monitoring across the Arab population. For these reasons, this study aims to validate the Arabic version of the SSGS 10-item Guilt and Shame scale. It is hypothesized that the Arabic 10-item SSGS will replicate the original scale’s two-factor structure for guilt and shame, showing satisfactory reliability and validity.

## Methods

### Minimum sample size

We estimated a minimum sample of 30–200 participants based on the recommendation of 3–20 times per scale’s variables (Mundfrom et al., 2005).

### Participants and procedure

In this cross-sectional study, an online survey was developed to collect data from participants using a snowball sampling method. The survey link was disseminated through social media platforms and personal networks (e.g., WhatsApp, Facebook, and email) to reach a wider audience. Participants were invited to complete the survey and encouraged to share it with their family, friends, and acquaintances. All participants provided informed consent for their participation prior to filling the survey. For the inclusion criteria: individuals have to be 18 years of age or older, currently living in Lebanon, Jordan, Egypt or Iraq, have a stable internet connection and willing to complete the Survey online. Those who did not meet the above inclusion criteria were excluded. The survey instruments were presented in a randomized way to limit order effects, and all the questionnaire items were set as mandatory in the Google Forms, preventing submission of incomplete responses. Participation was voluntary, anonymous, and uncompensated. Internet protocol (IP) addresses were monitored to prevent duplicate submissions. The study sample consisted of 611 adults (mean age = 21.85 ± 3.66 years, range 18–80) residing in Lebanon, Jordan, Egypt, or Iraq. The participants were a majority of females (74.9%), primarily single (96.6%), and had completed their education at the university level (95.9%). Other sample characteristics are presented in Table 1.

**Table 1:** Characteristics of the sample (*n* = 611).


Age (years)	21.85 ± 3.66 [min = 18; max = 80]
Household crowding index (person/room)	1.46 ± 1.11
Gender	
Male	154 (25.1%)
Female	460 (74.9%)
Country	
Lebanon	131 (21.3%)
Jordan	175 (28.5%)
Egypt	126 (20.5%)
Iraq	182 (29.6%)
Marital status	
Single	593 (96.6%)
Married	21 (3.4%)
Education	
Secondary or less	25 (4.1%)
University	589 (95.9%)

### Translation procedure

The State Shame and Guilt Scale was translated into Arabic following a rigorous forward-backward translation procedure in line with Beaton’s guidelines (Beaton et al., 2000). The original English version was first independently translated into Arabic by a professional Lebanese translator with no affiliation to the study. The Arabic version was translated back into English by another Lebanese psychologist fluent in English, therefore maintaining independence from the initial translation. Afterward, the original and back-translated versions were compared in detail, and any differences in wording or meaning were discussed and resolved by the research team and both translators (Fenn et al., 2020). Moreover, for cultural acceptance and conceptual equivalence, the Arabic version was checked for clarity, accuracy of language, and ease of understanding (Ambuehl and Inauen, 2022). Importantly, no cultural modifications to the original items were made, and the content was preserved to maintain equivalence with the original scale. Finally, a pilot study with 20 participants confirmed their understanding of all the items, and no changes were necessary.

### Measures

State Shame and Guilt Scale (SSGS): The SSGS is used to evaluate both shame and guilt (Marschall et al., 1994). Each of the emotional experiences, guilt and shame, are equally evaluated: guilt is measured through items 2, 4, 6, 8, and 10 (e.g., “I feel bad about something I have done”) and shame using items 1, 3, 5, 7, and 9 (e.g., “I feel like I am a bad person”). In general, the shame subscale measures feelings of inferiority, humiliation, and how the person sees himself badly, while the guilt subscale measures the actual act of doing something bad and the need to correct or make amends for it. Each participant completes each question using a 5-point Likert scale, which has descriptive labels from 1 (“Not feeling this way at all”) through 5 (“Feeling this way very strongly”). The responses are summed together to form a total score between 5 and 25, with higher scores reflecting a more intense feeling of guilt or shame.

Patient Health Questionnaire (PHQ-4): Validated in Arabic, the PHQ-4 is a self-report scale to assess the level of psychological distress experienced by an individual (Obeid et al., 2024). It is a concise instrument with 4 items to capture symptoms of anxiety (e.g., “Feeling nervous, anxious, or on edge”) and depression (e.g., “Little interest or pleasure in doing things”) over a period of two weeks. The 4 items present in the scale are divided equally between anxiety and depression, 2 items for each subscale. Responses are recorded using a 4-point Likert scale, with scores ranging from 0 (“not at all”) to 3 (“nearly every day”). The overall psychological distress score is obtained by adding all the responses of the 4 items present in the scale. A score of 3 or higher indicates clinically relevant symptoms of psychological distress. In fact, the level of distress can be assessed following the scale established intervals: scores of 0–2 reflect no psychological distress, 3–5 correspond to mild distress, 6–8 indicate moderate distress, and 9–12 represent severe psychological distress.

External and Internal Shame Scale (EISS): Validated in Arabic, the EISS is an eight-item self-report tool developed to measure experiences of shame from both interpersonal and intrapersonal perspectives (Fekih-Romdhane et al., 2023a). The scale focuses on assessing four main dimensions of shame: feeling inferior or inadequate, feeling socially excluded, feeling useless/empty inside, and being sensitive to judgment or criticism. Each of the dimensions is represented by two items, one for External Shame (ES) (e.g., “I feel that others see me as uninteresting”) and one for Internal Shame (IS) (e.g., “I feel that I am different and inferior to the others”), resulting in 4 items for ES and IS, totaling of 8 items. While filling the survey, participants rate their answers on a 5-point scale (0 = “Never,” 4 = “Always”). The responses are added together to yield a total score between 0 and 32, with a higher score meaning a higher level of shame experience.

Big Three Perfectionism Scale - Short Form (BTPS-SF): Validated in Arabic, the BTPS-SF (Fekih-Romdhane et al., 2023b) is a brief 16-item questionnaire designed to assess three types of perfectionism: rigid perfectionism (e.g., “I have a strong need to be perfect”), self-critical perfectionism (e.g., “The idea of making a mistake frightens me”), and narcissistic perfectionism (e.g., “I get frustrated when other people make mistakes”). The scale uses a 5-point Likert scale where the participants are asked to rate their agreement with each of the 16 items from “Strongly Disagree” to “Strongly Agree.” After completion, the total score is calculated by adding the responses together, with higher scores indicating a greater level of perfectionism in each area.

### Analytic strategy

No missing data was found in the database since all questions were required in the Google Forms. We used data from the entire sample to perform a confirmatory factor analysis (CFA) using RStudio (R version 4.5.2) with the “lavaan” and “SemTools” packages (Jorgensen et al., 2012; Rosseel et al., 2014). Given the ordinal nature of the items, the Weighted Least Squares Mean and Variance Adjusted (WLSMV) estimator was used, which is considered the most appropriate for ordinal data. Multiple fit indices were calculated: root mean square error of approximation (RMSEA) (≤ 0.08), standardized root mean square residual (SRMR) (≤ 0.05), Tucker-Lewis Index (TLI), and Comparative Fit Index (CFI) (≥ 0.90 for both) (Byrne, 2013). Additionally, convergent validity was checked via the average variance extracted (AVE) ≥ 0.50. Mardia’s test skewness and kurtosis values confirmed the non-multivariable normality.

A multi-group CFA was conducted to examine measurement invariance of shame and guilt scores between genders and countries (Chen, 2007) at the configural, metric, and scalar levels (Vandenberg and Lance, 2000). ΔCFI ≤ 0.010 and ΔRMSEA ≤ 0.015 or ΔSRMR ≤ 0.010 supported the evidence of invariance.

Internal reliability was assessed using McDonald’s *ω* and Cronbach’s *α*, with values greater than 0.70 reflecting adequate composite reliability.

To assess construct validity, convergent and discriminant validity were examined through Spearman correlation analyses between shame and guilt scores and theoretically related psychological constructs. Specifically, correlations were computed with internal and external shame (EISS), psychological distress (PHQ-4), and perfectionism (BTPS-SF). Convergent validity was supported by positive associations between shame and guilt and related constructs such as internal/external shame and psychological distress. Additionally, correlations with perfectionism were examined as further evidence of construct validity. Spearman’s rank correlation coefficients were used given the ordinal nature of the data and the non-normal distribution of the variables. Comparison of shame and guilt scores between genders was done using the Mann–Whitney test and between countries using the Kruskal-Wallis test.

## Results

Participants’ characteristics are summarized in Table 1.

### Confirmatory factor analysis

**Figure 1:**
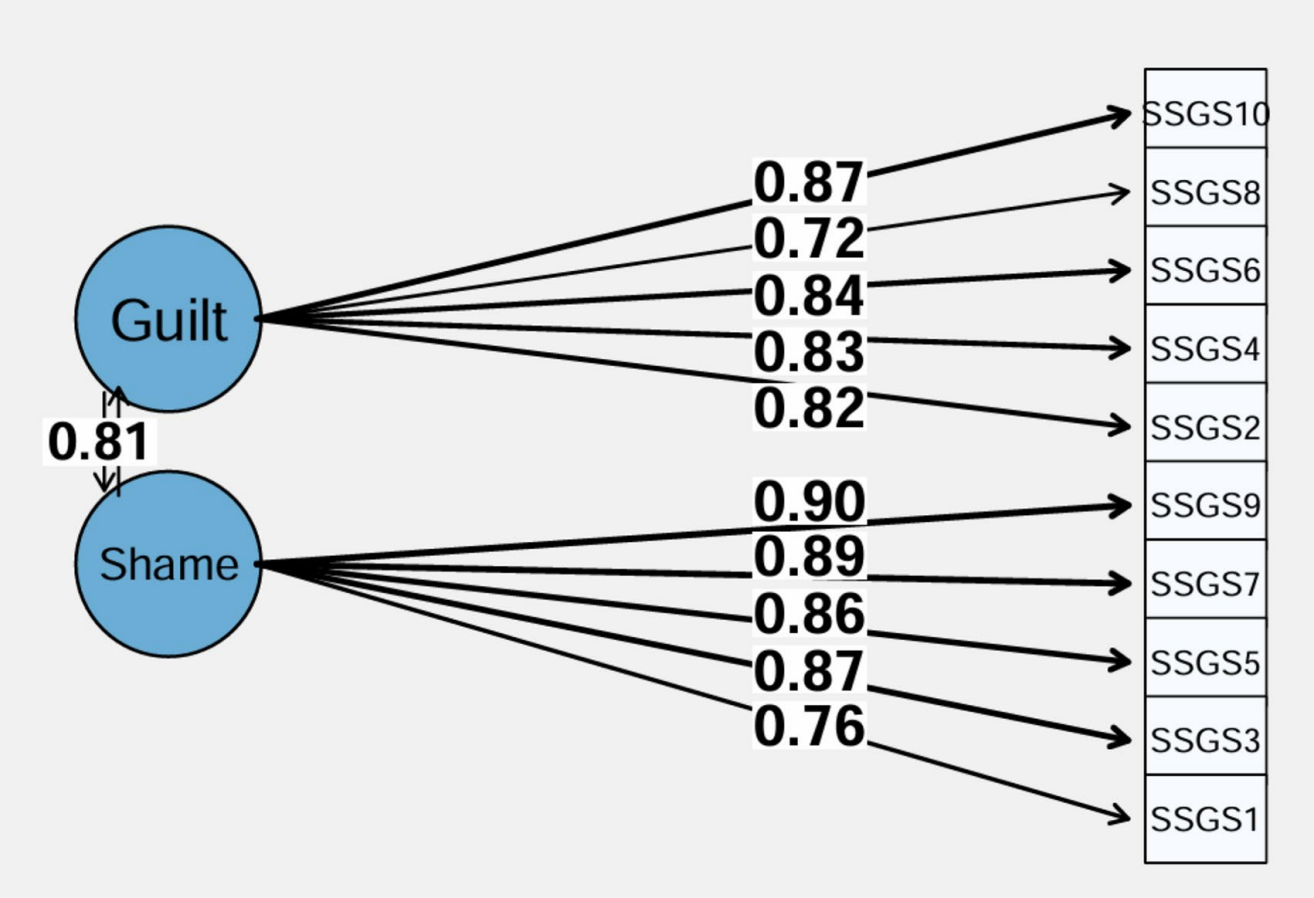
Standardized loading factors deriving from the confirmatory factor analysis of the two-factor model of the State Shame and Guilt Scale.

The fit indices of the two-factor model were acceptable (X^2^(34) = 175.62, *p* < 0.001, RMSEA = 0.082 (90% CI 0.071, 0.095), SRMR = 0.059, CFI = 0.993, TLI = 0.991). Standardized factor loadings were excellent (Figure 1). Internal reliability was adequate for the shame (ω = 0.86/α = 0.87) and guilt (ω = 0.87 / α = 0.87) scales. The AVE value (= 0.75) was satisfactory.

### Measurement invariance

Regarding genders, invariance was supported at all levels (Table 2, Model 1), with no difference found between males and females in terms of guilt (Median = 11; IQR = 8 vs. Median = 10; IQR = 7.75, Mann–Whitney U = 33787.50, Z = −0.86, *p* = 0.389) and shame (Median = 7; IQR = 6 and Median = 7; IQR = 5, Mann–Whitney U = 34,460, Z = −0.52, *p* = 0.605) scores.

**Table 2:** Measurement invariance of the shame and guilt across gender and countries.

Model	χ^2^	df	CFI	RMSEA	SRMR	Model comparison	ΔCFI	ΔRMSEA	ΔSRMR
Model 1: Across gender									
Male (single-group CFA)	76.865	34	0.993	0.091	0.072				
Female (single-group CFA)	12.445	34	0.994	0.079	0.061				
Configural	209.311	68	0.994	0.082	0.064				
Metric	221.658	76	0.993	0.079	0.065	Configural vs. metric	0.001	0.003	0.001
Scalar	224.936	96	0.994	0.066	0.064	Metric vs. scalar	0.001	0.013	0.001
Strict	237.956	106	0.994	0.064	0.065	Scalar vs. strict	<0.001	0.002	0.001
Model 2: Across countries									
Lebanon (single-group CFA)	90.123	34	0.992	0.113	0.098				
Jordan (single-group CFA)	69.730	34	0.995	0.078	0.063				
Egypt (single-group CFA)	56.551	34	0.996	0.073	0.076				
Iraq (single-group CFA)	83.409	34	0.990	0.090	0.081				
Configural	286.143	136	0.993	0.085	0.081				
Metric	326.775	160	0.992	0.083	0.087	Configural vs. metric	0.001	0.002	0.006
Scalar	315.702	190	0.994	0.066	0.082	Metric vs. scalar	0.002	0.017	0.005
Strict	373.578	220	0.993	0.068	0.088	Scalar vs. strict	0.001	0.002	0.006

χ^2^ = chi-square statistic; df = degrees of freedom; CFI = Comparative fit index; RMSEA = root mean square error of approximation; SRMR = Standardized root mean square residual. ΔCFI, ΔRMSEA, and ΔSRMR represent changes in fit indices between nested models. Measurement invariance was considered supported when ΔCFI ≤ 0.010, ΔRMSEA ≤ 0.015, and/or ΔSRMR ≤ 0.010.

Invariance was also supported across countries (Table 2, Model 2). No significant difference between countries was found in terms of shame (Lebanon (Median = 7; IQR = 4), Jordan (Median = 7; IQR = 6), Egypt (Median = 7; IQR = 7) and Iraq (Median = 7; IQR = 4), Kruskal-Wallis H = 4.76, *p* = 0.191) and guilt (Lebanon (Median = 9; IQR = 10), Jordan (Median = 11; IQR = 8), Egypt (Median = 11; IQR = 7) and Iraq (Median = 10; IQR = 8), Kruskal-Wallis H = 4.21, *p* = 0.239) scores.

### Concurrent and discriminant validity

Higher shame and guilt scores were associated with higher perfectionism, internal and external shame, and psychological distress scores (Table 3).

**Table 3:** Correlation matrix between scores.

	1	2	3	4
1. Shame	1			
2. Guilt	0.67***	1		
3. Internal and external shame	0.61***	0.48***	1	
4. Psychological distress	0.45***	0.38***	0.40***	1
5. Perfectionism	0.40***	0.40***	0.49***	0.31***

^***^p < 0.001. Numbers in the table refer to Spearman coefficients (rho).

## Discussion

The current study was conducted to establish the validity of an Arabic version of the SSGS. Overall, our results demonstrate that the Arabic scale has a clear two-factor structure, strong reliability, and good evidence for concurrent validity. Furthermore, these results suggest that the scale operates similarly across gender and national groups, supporting its validity for both cross-group and cross-country comparisons. Our results affirm that the Arabic SSGS is a brief, readable, and practical self-report instrument for research purposes. Therefore, it supports the satisfactory reliability and validity of the assessment of guilt and shame within the Arabic-speaking population. Importantly, our findings should be interpreted within the context of a predominantly young and highly educated sample, which may limit generalizability to older, less educated, or clinical populations.

Our study strongly supports a two-factor model of shame and guilt, with acceptable-to-good fit indices from confirmatory factor analysis. Despite the RMSEA value of 0.082, which is slightly above the most conservative thresholds, it remains acceptable overall, particularly given our high CFI/TLI. In addition, the chi-square test was statistically significant, as expected in large samples such as our own. The excellent standardized factor loadings also provide further evidence for the structural validity of the two-factor model compared with previous validation efforts. These results support the distinction between shame and guilt as related but separate self-conscious emotions (Tangney and Dearing, 2003). This pattern aligns with prior validation studies conducted in non-Arabic populations (Cavalera et al., 2017; Tzelepi et al., 2022). In fact, the CFA resulting from the Italian validation (Cavalera et al., 2017) indicated an adequate model fit, but our analysis showed that the fit indices, specifically our CFI and TLI, were of higher quality. Thus, our study reflects a stronger correlation between the measured items and the emotion they are intended to assess. The Greek validation (Tzelepi et al., 2022) used a three-factor structure (pride, shame, and guilt) based on exploratory factor analysis, which is also consistent with the original concept of the scale. However, because they used an EFA, we cannot directly compare their results to our study’s CFA. Nonetheless, our data indicate that even when pride is removed from the analysis, the distinction between shame and guilt is maintained. The replication of these results in our Arabic-speaking sample suggests that the 10-item brief self-report measures evaluate these emotions reliably across cultural contexts.

Furthermore, both subscales showed excellent internal reliability, with McDonald’s omega values (*ω*) of approximately 0.86 for shame and 0.87 for guilt. In contrast to previous validation studies (Tzelepi et al., 2022), our analysis shows that the Arabic 10-item SSGS has stronger psychometric properties. While the Greek validation reported acceptable internal consistency for shame (*α* = 0.717) and guilt (α = 0.770), our study’s reliability estimates were higher for both subscales, with very good internal consistency, Cronbach’s α ≥ 0.86 for both emotions. In addition, if we compare our results with the original validation study (Marschall et al., 1994), our data show slightly higher values, indicating that each subscale reliably measures the intended self-conscious emotion, supporting the scale’s use in research. Multi-group CFA showed minor changes in CFI and RMSEA across the constrained models, indicating configural, metric, and scalar invariance across genders and among participants from Lebanon, Jordan, Egypt, and Iraq. This showed that participants understood and responded to the items in a comparable manner. As such, it can be inferred that the obtained differences in guilt or shame scores between the groups likely reflect real differences rather than measurement bias. Thus, the collected data may be helpful in providing a reliable tool for cross-cultural and cross-gender research in the Arab region, where consistent measurement tools are limited.

In addition, the pattern of associations regarding external variables serves as a clear indicator of concurrent validity. The correlation analysis carried out in our study found strong associations between shame and guilt (*p* = 0.67), along with moderate correlation with internal and external shame, psychological distress, and perfectionism. Firstly, evidence suggests that psychological distress, shame, and guilt are closely tied together (Kim et al., 2011; Oasi et al., 2025). This finding is supported by our data, where psychological distress is moderately correlated with shame (*p* = 0.45) and guilt (*p* = 0.38). Thus, this implies that self-conscious emotions have an association with mental health outcomes among Arabs. Therefore, individuals with high levels of shame tend to evaluate themselves negatively, which may contribute to greater levels of anxiety, depression, and psychological stress. Although guilt focuses more on individual behavior, it still results in an emotional burden, but to a smaller degree than shame. Secondly, our data showed that perfectionism has a modest correlation with shame, guilt, internal and external shame, and psychological distress, with p ranging from 0.31 to 0.49. Thus, perfectionism may potentially accentuate the negative effect on self-consciousness when associated with these factors. A person with a maladaptive form of perfectionism, characterized by a high level of self-criticism and performance standards, might experience greater feelings of shame and guilt, along with psychological distress symptoms. Our results align with the current literature suggesting that perfectionism is associated with heightened emotional vulnerability in people prone to self-evaluation (Barritt, 2017; Stoeber et al., 2007). Finally, understanding how these factors are interrelated can enhance our understanding of psychological functioning and provide an explanation for why certain people show higher levels of anxiety and depression than other individuals. This study contributes to current research by examining how individuals view their self-conscious emotions, mental health, and personality characteristics. This offers a better model for understanding the relationship among them.

### Clinical implications

This study advances psychological assessment by providing professionals with a validated and culturally appropriate tool for evaluating guilt and shame in Arabic-speaking populations. The results support the inclusion of both guilt and shame as important targets in the Arab region, rather than considering them as background emotions with no significant impact on an Arab’s mental health. The positive associations between guilt and shame with psychological distress suggest that these constructs are meaningfully related in this population and could be relevant for further clinical research and screening contexts. For instance, individuals presenting with psychological distress often exhibit increased levels of guilt and shame. This highlights the importance of evaluating these two types of emotions using a psychometrically validated Arabic scale. Furthermore, efforts to validate the 10-item SSGS in its Arabic version demonstrated reliable performance across all groups, as evidenced by measurement invariance at multiple levels. This indicates that the scale has the potential to serve as a reliable measurement tool for use across diverse Arabic-speaking populations in research and comparative studies. Lastly, the scale’s concurrent validity, showing that guilt and shame can coexist with perfectionism, internal and external shame, and psychological distress, suggests it may assist experts to better understand patients’ well-being through descriptive emotional patterns rather than diagnostic interpretations. Given the cross-sectional nature of the study, these findings do not allow conclusions regarding clinical decision-making or treatment indications. In addition, several psychometric limitations should be considered when interpreting the clinical applicability of the Arabic SSGS. First, discriminant validity was not evaluated, limiting our ability to confirm that the scale clearly differentiates shame, guilt, and related constructs such as general distress or anxiety. Although convergent validity was supported, higher scores might reflect broader negative affect rather than specific self-conscious emotions. Therefore, in clinical practice, the scale should be used cautiously and ideally complemented with additional assessment tools. Second, the lack of test–retest reliability data prevents us from drawing conclusions about the measure’s consistency over time. Although the SSGS is designed to capture emotions related to the current state, such data are necessary to distinguish real emotional change from measurement variability. This also restricts its current use for tracking progress over time or assessing treatment outcomes. Overall, despite strong internal consistency and structural validity, the Arabic SSGS should be used primarily for cross-sectional research and initial clinical screening rather than for diagnosis or tracking changes over time. Therefore, longitudinal studies are needed to evaluate the scale’s sensitivity to change and potential utility for monitoring outcomes.

### Limitations

The current study has some limitations. First, we used a cross-sectional design and relied mainly on self-reported information, which makes it difficult to establish causal relationships and renders the study susceptible to response biases such as social desirability. Second, the participants were recruited from convenience samples from only four Arab countries, which may not reflect the whole diversity of the Arab region. Moreover, the participants were relatively young (mean age = 21.85 years), female (74.9%), highly educated, and recruited online. Therefore, caution should be exercised when attempting to generalize these research findings to the broader Arabic-speaking community, and our conclusions should be interpreted as applying primarily to a young, educated, and non-clinical population. Despite the scale being validated in many languages, the Greek validation provides the most comparable published data. While validation of the Italian and Tamil versions is available, limitations in comparability were noted. However, it should be noted that our study still marks an important step toward adapting a culturally appropriate psychometric measure for guilt and shame within the Arabic community. Finally, important psychometric properties, such as discriminant validity and test–retest reliability were not empirically assessed in the context of our study, which did not allow to test whether the Arabic SSGS is sensitive to change or if it can adequately capture situational fluctuations.

## Conclusion

The present study confirms that the Arabic version of the 10-item SSGS is both reliable and valid. The scale shows a clear two-factor structure and strong internal consistency, as well as cross-gender and cross-national measurement invariance, supporting its validity. Moreover, it shows a theoretically consistent association with various mental health outcomes. The effort of this validation will inspire local and international investigations into both self-conscious emotions. Having access to the Arabic form of the 10-item SSGS may be useful to researchers and clinicians working in this region. Finally, future research could verify our findings by testing the scale in other Arabic nations and by examining changes in guilt and shame over time using longitudinal designs.

## Data Availability

The original contributions presented in the study are included in the article/supplementary material, further inquiries can be directed to the corresponding author.
